# Long-Term Monitoring of Dzanga Bai Forest Elephants: Forest Clearing Use Patterns

**DOI:** 10.1371/journal.pone.0085154

**Published:** 2013-12-26

**Authors:** Andrea K. Turkalo, Peter H. Wrege, George Wittemyer

**Affiliations:** 1 The Wildlife Conservation Society, Africa Program, Bronx, New York, United States of America; 2 The Elephant Listening Project, Bioacoustics Research Program, Cornell Lab of Ornithology, Ithaca, New York, United States of America; 3 Department of Fish, Wildlife and Conservation Biology, Colorado State University, Fort Collins, Colorado, United States of America; 4 Save The Elephants, Nairobi, Kenya; Max Planck Institute for Evolutionary Anthropology, Germany, Germany

## Abstract

Individual identification of the relatively cryptic forest elephant (*Loxodonta cyclotis*) at forest clearings currently provides the highest quality monitoring data on this ecologically important but increasingly threatened species. Here we present baseline data from the first 20 years of an individually based study of this species, conducted at the Dzanga Clearing, Central African Republic. A total of 3,128 elephants were identified over the 20-year study (1,244 adults; 675 females, 569 males). It took approximately four years for the majority of elephants visiting the clearing to be identified, but new elephants entered the clearing every year of the study. The study population was relatively stable, varying from 1,668 to 1,864 individuals (including juveniles and infants), with increasingly fewer males than females over time. The age-class distribution for females remained qualitatively unchanged between 1995 and 2010, while the proportion of adult males decreased from 20% to 10%, likely reflecting increased mortality. Visitation patterns by individuals were highly variable, with some elephants visiting monthly while others were ephemeral users with visits separated by multiple years. The number of individuals in the clearing at any time varied between 40 and 100 individuals, and there was little evidence of a seasonal pattern in this variation. The number of elephants entering the clearing together (defined here as a social group) averaged 1.49 (range 1–12) for males and 2.67 (range 1–14) for females. This collation of 20 years of intensive forest elephant monitoring provides the first detailed, long term look at the ecology of bai visitation for this species, offering insight to the ecological significance and motivation for bai use, social behavior, and threats to forest elephants. We discuss likely drivers (rainfall, compression, illegal killing, etc.) influencing bai visitation rates. This study provides the baseline for future demographic and behavioral studies of this population.

## Introduction

African forest elephants (*Loxodonta cyclotis*), whose range includes the dense lowland forests of West and Central Africa, are estimated to comprise 25%-33% of the total African elephant population [1]. Genetic studies have proven that forest elephants are evolutionarily distinct from their savanna counterparts to a degree that merits distinct species consideration [2], although the International Union for the Conservation of Nature (IUCN) has not as yet recognized species status. Regardless, it is clear that forest elephants are of high conservation interest because of their ecological [3] and genetic distinctiveness.

The uniqueness of forest elephants is particularly salient in relation to the threats this species faces, with recent evidence from dung based surveys [1,3] and carcass counts [4] indicating large scale decline of forest elephant populations across their range. Studies of this species density that began in the 1980’s using dung counts and feeding trails [5-8], have been followed more recently by systematic transect surveys of dung density [1] and satellite telemetry [9-14]. Studies based on individual identification with sufficient data to assess visitation behavior at clearings have been few [12,15] and no studies have been able to estimate mortality, natality, or other demographic indicators for a forest elephant population. We therefore lack a basic knowledge of the demography and behavior of this species, one of the largest extant terrestrial vertebrates on the planet. This dearth of knowledge limits our understanding of its conservation status and ability to develop comprehensive conservation strategies and management programs. 

Known as “bais” in the local Babenzélé language, naturally occurring forest clearings provide an unparalleled opportunity to view wildlife and offer the primary means for direct observation and collection of demographic, ecological and behavioral data on forest species. While these bais vary in surface area, most are characterized by a groundcover of monocotyledons and are traversed by a water source [16]. Elephants are the most effective “architects” of bais, maintaining and increasing their surface area through the excavation of drinking pits, feeding activities, soil compaction, and digging for minerals or clay soils [17,18]. Well known by local people, bais traditionally provided hunting venues, but many have been depleted of wildlife with the advent of modern firearms and a bourgeoning market for bush meat and ivory.

In the early 1990’s several studies were initiated at bais in the Dzanga-Ndoki National Park (Central African Republic) and Nouabale-Ndoki National Park (Republic of Congo) [19]. These two parks, together with Lobeke National Park (Republic of Cameroon) make up the Sangha-Trinational area, a UNESCO World Heritage site. The Dzanga Forest Elephant Study at Dzanga Bai, Central African Republic, was initially conceived to learn more about the ecology and social behavior of a cryptic and poorly known species and to provide some measure of protection through consistent observer presence. The sustained, longitudinal nature of the study allows it to fulfill these original goals but it can also inform conservation strategies for this increasingly impacted species. 

Observational studies of forest elephants at clearings pose unique opportunities but also unique challenges for the interpretation of data relevant to their ecology and demography. In a sense, the ‘study population’ (i.e. those individuals observed visiting the clearing) is a self-selected and unknown proportion of the ‘source’ population of elephants inhabiting the surrounding landscape. Furthermore, while forest elephants enter clearings at all times of day, observations have been made almost exclusively during daylight hours. Nonetheless, observational studies at clearings provide unprecedented insight to the behavior and demography of this species. In this paper we provide detailed information on how forest elephants utilize the Dzanga Bai, from which we infer population trends and age structure of the study elephants. These baseline data are critical for interpreting the significance of a single bai for the elephants that use it and, thereby, develop effective conservation and management strategies for forest elephants. 

## Methods

### Ethics Statement

Research permits for this work were provided by Le Ministre d’Etat à l’Enseignement Supérieur et à la Recherche Scientifique and the Dzanga-Sangha Project. This entirely observational study was conducted in a protected area, the Dzanga-Ndoki National Park.

### Study Area

**Figure 1:**
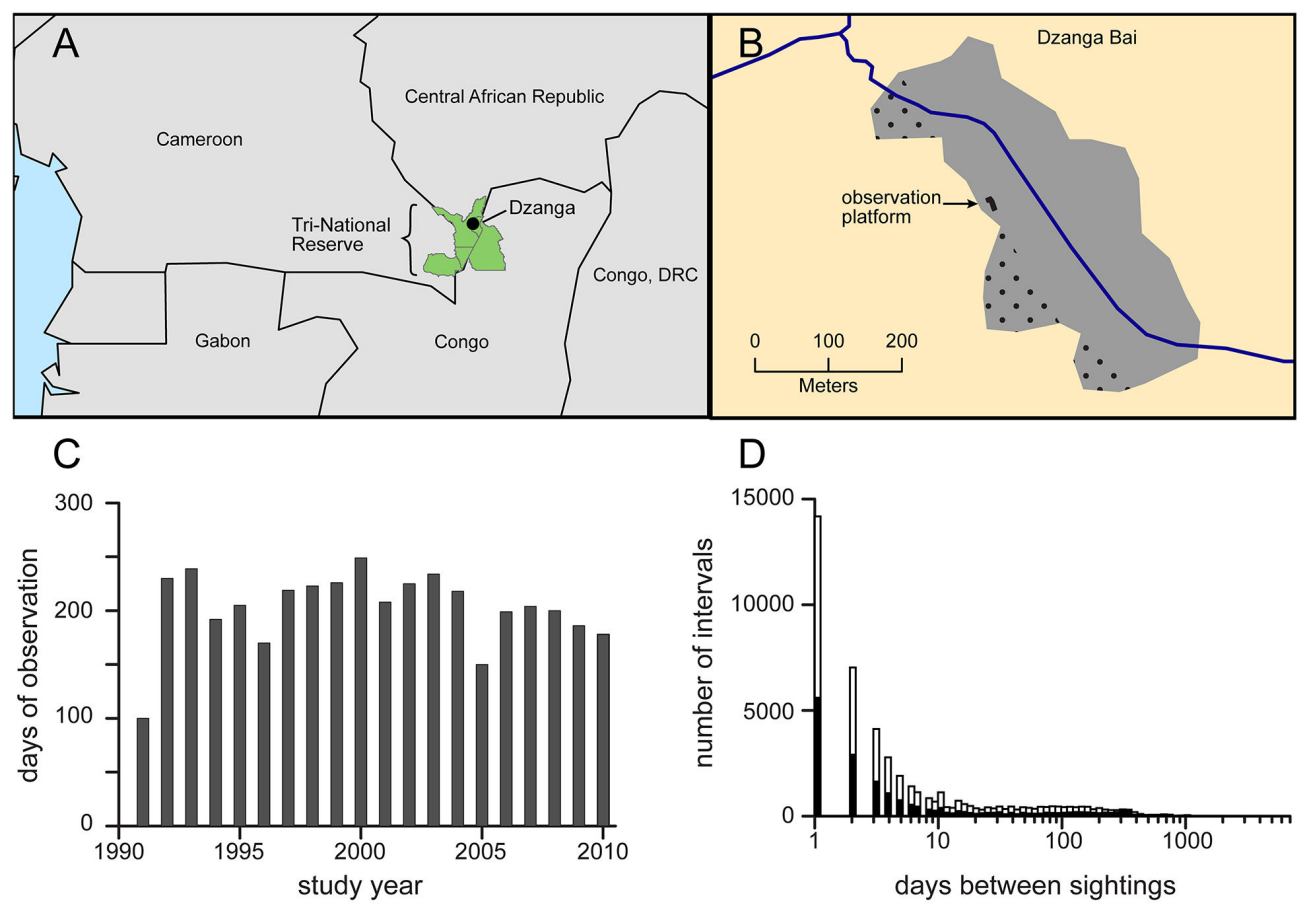
The Dzanga Bai, annual observation effort, and sighting intervals for identified elephants. (A) The location of Dzanga Bai in Central Africa. (B) Dzanga bai, showing the location of the observation platform and bisecting stream. The stippled areas could not be seen from the platform. (C) Observation days per year during the 20-yr study. (D) The distribution of days between successive sightings of identified adults. For females (open bars; 49,235 intervals) median=3, mean=42, max=15.5 yrs. For males (black bars; 21,843 intervals) median=4, mean=64, max=16.8 yrs.

Dzanga Bai is located in southwestern Central African Republic in the Dzanga-Ndoki National Park. (2.963° N, 16.365° E) (Figure 1a). Measuring roughly 200 by 500 meters (10 hectares), this natural forest clearing consists of a sandy pan bisected by a permanent stream, the Dzanga (Figure 1b). During drier periods of the year (December-March) the bai surface dries out, facilitating the excavation of holes by elephants to access dissolved minerals. A basic description of Dzanga Bai, the surrounding vegetation, and general data collection methods are available in Turkalo and Fay [19]. Precipitation was recorded daily using a standard rain gauge located at the main research camp, 2 km from the bai. Rainfall averaged 1660 mm per year (range 1359 - 2055) and December through March were the months with lowest rainfall.

Daily observations were made with a spotting scope and binoculars from an elevated platform located on the western edge of the clearing, which is typically downwind of the elephants (Figure 1b). The 7m-high platform provided an unimpeded view of nearly the entire clearing. Observation sessions were conducted between 13:30 and 16:30-17:00 hrs, the time period when the largest numbers of elephants are visible in the bai. The average number of observation days per year was 203 (SD=34; Figure 1c). On average only two months per year had fewer than 10 days of observation, however these fell on different months each year such that all months were equally sampled over the 20-yr study.

### Individual Identification

All elephants that enter the bai are individually identified by distinctive ear pattern of tears and holes, curvature of tusks and estimated length, tail morphology, body scarring and any other features useful in re-identification [20,21]. For each individual, identity cards are created with simple line drawings of identifying features, gender, and age class. Photographic documentation is obtained when possible and has proved invaluable for refining estimates of age. From the beginning of the study, age-class was estimated from shoulder height, size relative to adults/mother, skin morphology, and other characteristics. Aging is highly accurate for younger individuals tracked since birth, but less exact for individuals first identified when over 25 years of age. In 2013 a formal aging rubric for forest elephants was published using data from known-aged individuals, shoulder height measurements (Laser Technology Criterion 400 rangefinder), comparison of replicated images for the same individuals over 20 years, and other relevant characteristics [21]. Here, the initial age-class designations of elephants first identified between 25-40 years old were confirmed or adjusted by evaluating their earliest photographs in comparison to the aging rubric. 

Individuals difficult to re-identify because they lacked distinctive features and/or were initially observed for only a short time were excluded from analyses. Our sample included only individuals seen over a span of at least nine months (n=3144 individuals, or 65% of all elephants individually recorded, where 68% of excluded animals were seen only a single time). For some analyses we further restricted the sample to a core set of best-known adults (n=840) satisfying the following criteria: (i) seen a minimum of 12 times; (ii) observed over three or more years, and (iii) with an average of at least four observations for each year they were alive. While these criteria are necessarily arbitrary, they provide an unbiased way to construct an analysis dataset that includes all of the best-known individuals. All offspring of this core set of adults were included in the sample (n=825). Individuals in this potential core set were excluded if they were difficult to identify, and a few families were excluded due to having multiple adults that were difficult to identify.

### Data Collection

All data were collected by AKT. Regular observations began in June 1990 and here we analyze data collected over 20 years, ending in May 2010. We define each “study year” as running from June through the following May (e.g. 1 June 1990-31 May 1991 = study year 1991). At the start of observations each day, known individuals already present were noted. During an observation session, the time, entrance location, group size, and identities of all elephants entering the bai were recorded. Because forest elephants are difficult to observe within the forest, the size of entering groups is typically used to estimate the basic, or nuclear, group size of this species [22,23]. In the case of unknown individuals the gender and age class were recorded and identity cards created. The total number of elephants in the bai was tracked using scan counts conducted every 30 minutes. Because elephant numbers tend to increase through the afternoon and into the night [19,24], but occasionally a stampede can entirely empty the bai, we used the maximum count from any scan on a particular day in analyses. We omitted days when the latest count was made before 16:00 hrs (2.5% of days with counts omitted).

Individual elephants and family groups are typically observed in the bai on several contiguous days or for a bout of days with short absences. These bouts are separated by much longer periods of absence, often many months or even years. Radio-telemetry studies have shown that these longer absences often involve dispersal over distances of many kilometers [25]. In order to capture the pattern of visitation over time scales of months and years, strings of observations separated by no more than six days of absence (twice the median observation interval, Figure 1d), were lumped together as a single elephant '*visit*' with return after an absence of six days considered a new visit. 

In order to evaluate whether there were ‘resident’ and ‘nonresident’ families at Dzanga (sensu [26]) we plotted the average number of months per year individual elephants were observed in the bai. The years in which an individual was first and last observed were omitted because they would bias the ‘average months per year’ statistic.

We used two approaches to track changes in overall population size across the 20 years of study: number of individuals observed adjusted by observation effort (proportion of observation days per year) and the number of individuals estimated to be alive per year. We assigned date of death based on (1) observed carcasses of known elephants (rare), (2) very young calves missing when observing their known mother (rare), and (3) individuals not seen for four times their inter-visit interval, a protocol adapted from Wittemyer [27]. Individuals from the latter category were assigned a death date of twice their inter-visit interval 

### Statistical Analysis

All statistical analyses were run using SAS^®^9.2 (Carey, NC, USA) and tests were considered significant at an experiment-wise error rate of 0.05.

We used standard methods to test departures of sex ratio and counts of elephant density in the clearing against expected distributions. The observed sex ratio was tested against an expected binomial proportion of 0.5. Possible change in the sex ratio over the course of the study was evaluated using the Cochran-Armitage test for a trend among binomial proportions, with the null hypothesis being no trend. We compared the distribution of visits by males and females to the random Poisson distributed expectation using a Kolmogorov-Smirnov two-sample test, which is sensitive to differences in location as well as dispersion.

Change in the age-class distribution of males and females was examined by comparing the distribution early in the study, once most of the core individuals were identified (1995) and late in the study (2010). A Chi-square statistic was used to test the null hypothesis that frequencies across age-classes were similar across the two periods for each sex.

We investigated the relationship between rainfall and the number of elephants visiting the Dzanga clearing in two ways. Previous studies of forest elephant clearings have suggested basic differences in visitation between wet and dry periods of the year [12,22,28]. For comparative purposes we used a General Linear Model (GLM) approach to evaluate whether the average number of elephants in the clearing differed according to season. In a study spanning 20 years, it is not surprising that annual wet and dry seasons vary in their intensity. Therefore, we examined the influence of rainfall at a finer scale by correlating average monthly densities of elephants with total monthly rainfall (as well as investigating one- and two-month lag variables for rainfall).

## Results

**Figure 2:**
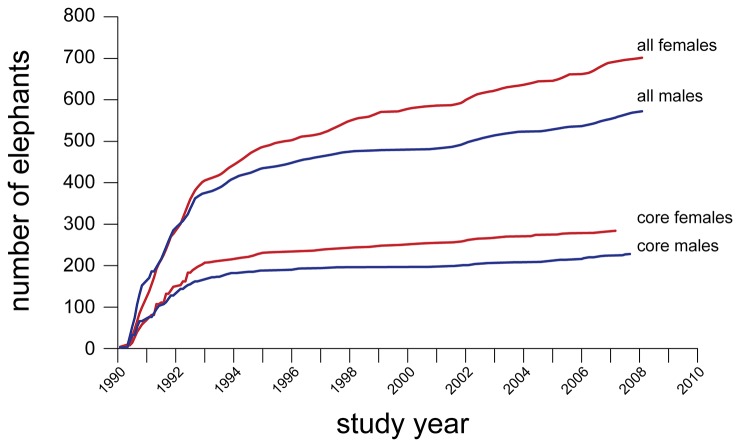
Cumulative number of uniquely identified adult elephants through 20 years of study. All elephants in this analysis were entered as adults and were seen over at least 9 months, core elephants were seen more frequently (see methods).

Over the course of 20 years of observation, 3144 forest elephants were uniquely identified. An additional 1851 elephants were identified but seen too rarely to be confident of their identity (and were subsequently excluded from all analyses). Excluding maturing offspring, 676 adult females and 569 adult males were identified in the clearing over the course of the study. The frequency with which individuals visited the clearing was highly variable (Table 1) and as a result it took approximately four years of near continuous observation to identify the Dzanga population (cumulative identification rate visually reaching an asymptote, Figure 2). New adults of both sexes (and associated offspring) continued to enter the bai population throughout the study, though the rate slowed after the first four years (Figure 2). From 1995 (when 76% of the best-known ‘core’ sample was known; Figure 2) until 2010, 983 new adults were identified. While slightly more than half of these appeared to be relatively transient, 40% were seen regularly in the years after identification. 

**Table 1:** Mean observation frequency of independent, known elephants entering Dzanga Bai^*1*^.

		**days btwn sightings^*2*^**		**days btwn visits^*2*^**		**days/visit**		**sightings/year**	
**Sex**	**Subcategory (n)**	**Mean**	**STD**	**Mean**	**STD**	**Mean**	**STD**	**Mean**	**STD**
Female	All (1489)	169	403	240	493	2	1	8	8
	Core (927	48	64	119	145	3	2	13	8
Male	All (1155)	144	338	222	432	2	1	6	6
	Core (712)	55	55	132	125	3	1	10	7

^1^ dependent offspring not included because their mother largely dictates their behavior.

^2^ ‘sightings’ refers to the number of days an individual was observed while ‘visit’ refers to a bout of sightings close in time (see methods).

**Figure 3:**
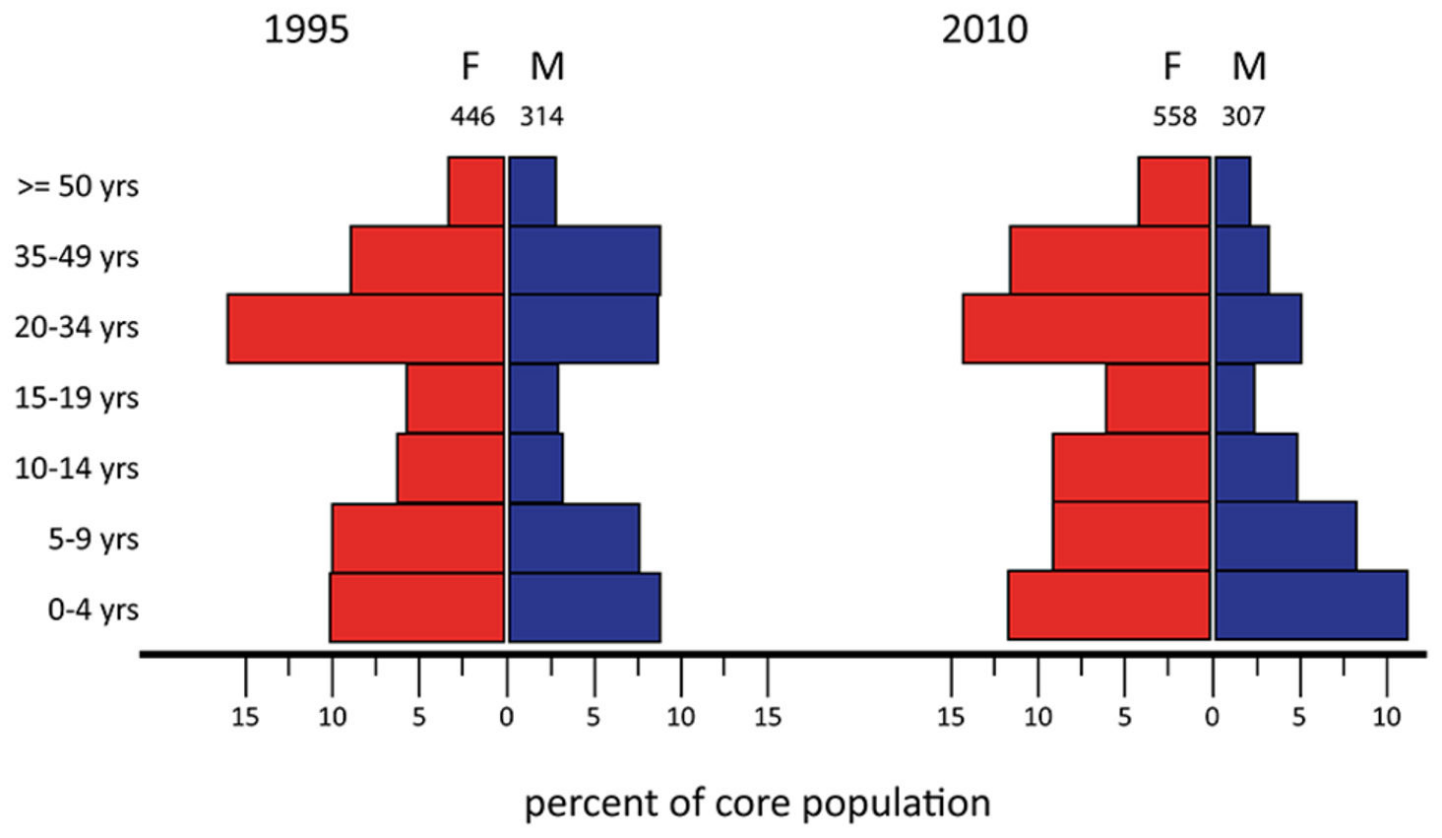
Changes in the sex and age-class distribution over a 15 year period. Mortality among adult males increased over the study period. Only the best-known (core) groups are included in the age pyramid as other, less known elephants were not aged. The proportion of the population in the ‘sub-adult’ age-classes (10-19 years of age) likely is biased low because of dispersal from the natal group before individually distinct characters allowing identification were developed.

The sex ratio was significantly skewed toward females in all years (test of binomial proportion, p<0.01), with an average ratio of 3:2 (females:males). However, the sex ratio skew became increasingly pronounced as the study progressed. In 1995, the sex ratio among identified elephants was 56% female. By 2010 the sex ratio was nearly 3:1 in favor of females (70% female) showing a consistent and significant trend across years (Cochran-Armitage Z=10.8, p<0.0001). A closer look at the age-class distribution for the core sample of individuals indicates that adult males accounted for a smaller proportion of the population by 2010 (Figure 3). For females, the distribution across age-classes did not differ significantly between the beginning and end of the study, but for males the change was significant (likelihood ratio chi-square = 9.1 p=0.15 and 41.0 p<0.01, respectively).

**Figure 4:**
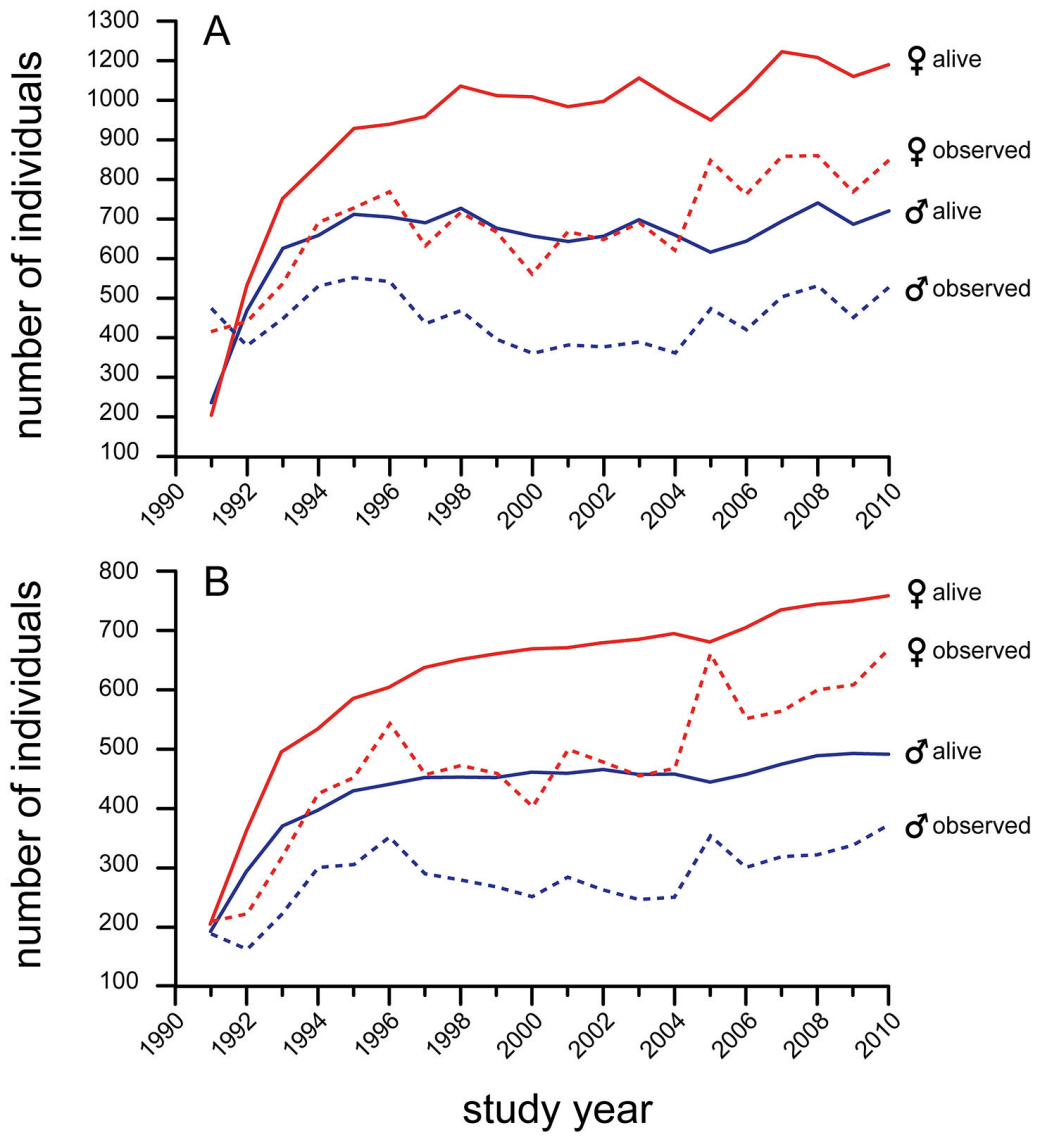
Annual size of the population visiting Dzanga Bai. (A) Estimates of the total population (individuals of all ages, including immigrants and those born into the population as of the end of each study year). (B) Estimates of the core population. Solid curves include all individuals known alive in that year, whether observed in the bai or not. Dashed curves include only individuals actually observed, adjusted for the number of observation days in each year.

Size of the study population (number of individuals known to be alive in a given year, even if that individual was not actually observed) remained remarkably stable after about 1994, when the majority of individuals had been identified (Figure 4). Similarly, the actual number of individuals observed adjusted for observation effort also demonstrated stability over the course of the study (Figure 4). 

### Visitation Behavior

**Figure 5:**
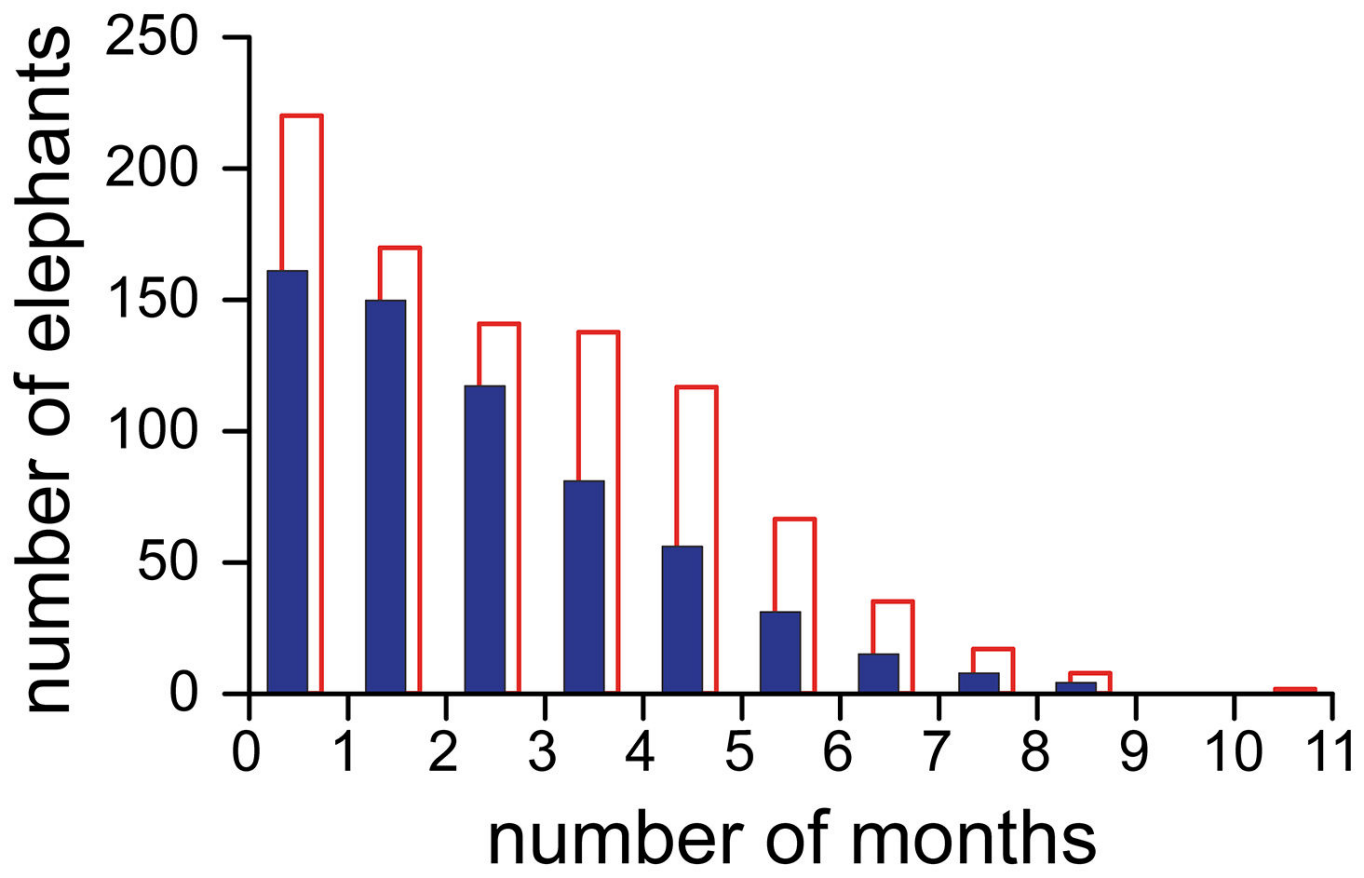
Average number of months per year that independent individuals enter the bai. The sample includes 914 females (open bars) and 623 males (solid bars). Only years during which the individual was known alive for the entire year are included (i.e. the year of initial identification and the year of death are omitted).

The patterns of visitation were highly variable between individual forest elephants and elephant families, but relatively consistent for a given group. On average families visited the bai for 2-3 visitation bouts per year, with each visitation bout averaging a few days (Table 1). However some families were frequently observed (weekly) while others, males in particular, might be absent for years at a time. The distribution of male and female visiting frequency departed significantly from random (poisson distribution, χ^2^ test, p<0.01), but did not demonstrate multiple modality (Figure 5). Males visited the bai significantly fewer times each year than females (Kolmogorov-Smirov 2-sample test, p<0.01).

**Figure 6:**
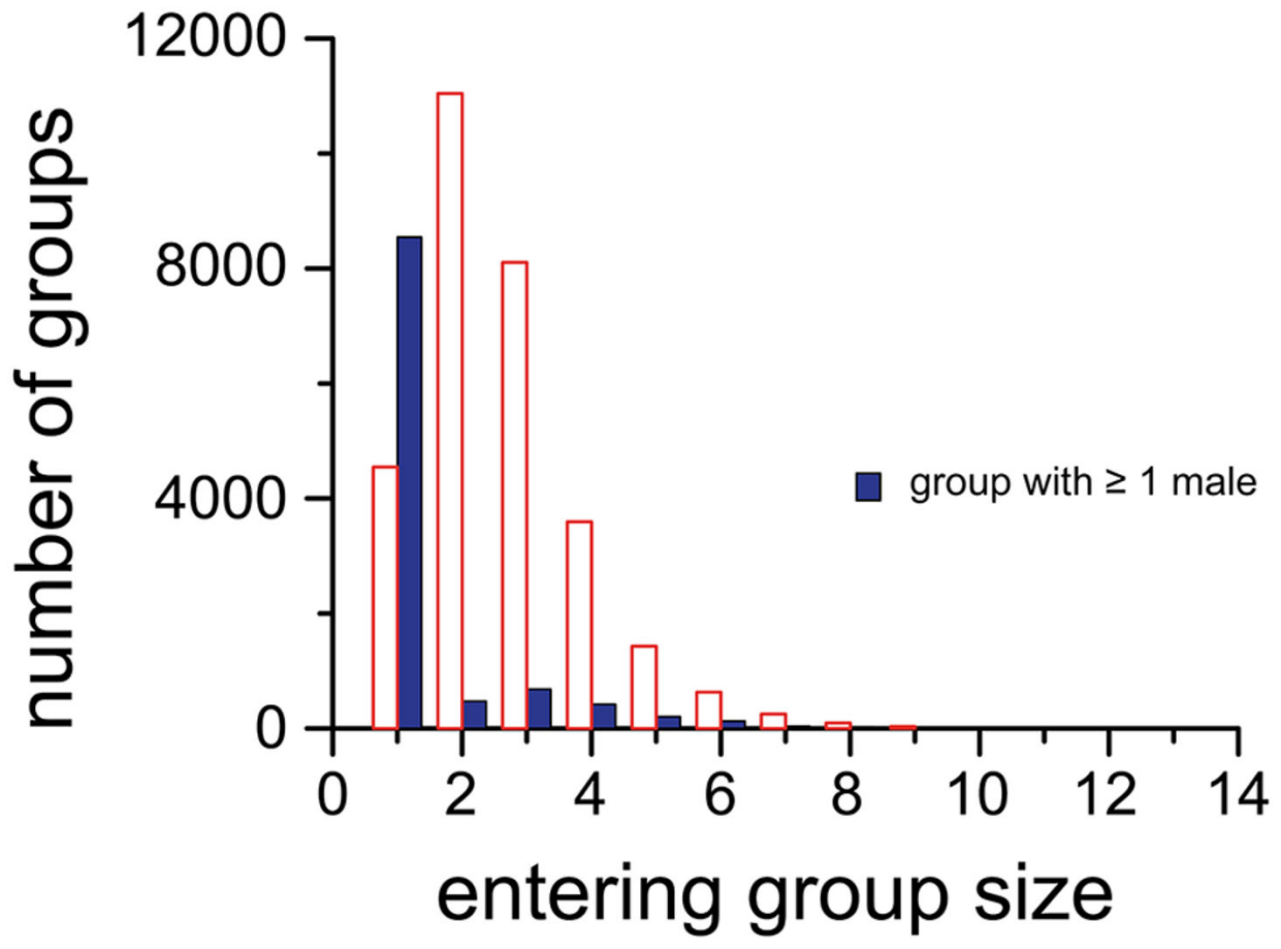
Size of forest elephant groups entering Dzanga Bai. Groups may include only females and dependent juveniles (open bars) or may include one or more males (solid bars).

The size and membership of 40,320 groups entering the bai were recorded. Groups ranged from a single individual to 14 individuals with a mean group size of 2.36 (std = 1.38) (Figure 6). Groups with one or more adult males (n=10,549) and those composed only of females or females and their dependent offspring were analyzed separately. Adult males entered the clearing alone 81% of the time. When entering with other elephants, a male was with females 90% of the time, most likely because the male was either mate-guarding or interested in a female in the group. All-male groups were rare, occurring in only 1% of groups that included a male. The average size of female groups was 2.7 (std = 1.3, n=29,771) and 80% of groups included three or fewer individuals. Excluding solitary females (15%), family groups averaged 3.0 individuals (std=1.2, n=25,223).

### Seasonality and elephant activity patterns

**Figure 7:**
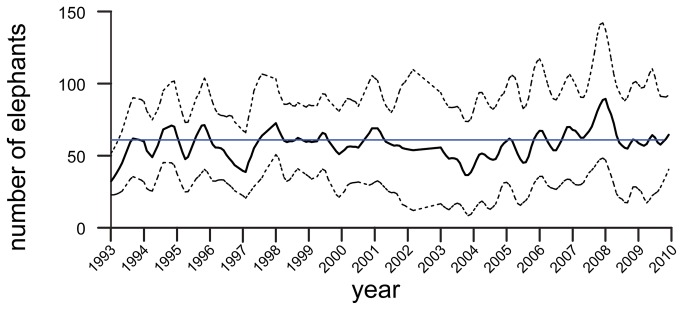
Variation in the number of elephants observed in the Dzanga clearing over 20 years. The mean daily value (solid curve), the minimum and maximum counts (dashed curves), and the mean monthly count across all years (light line) are shown. Curves were generated with a Loess smoother function (tension=0.1). Months with fewer than 10 days of observation were omitted.

**Figure 8:**
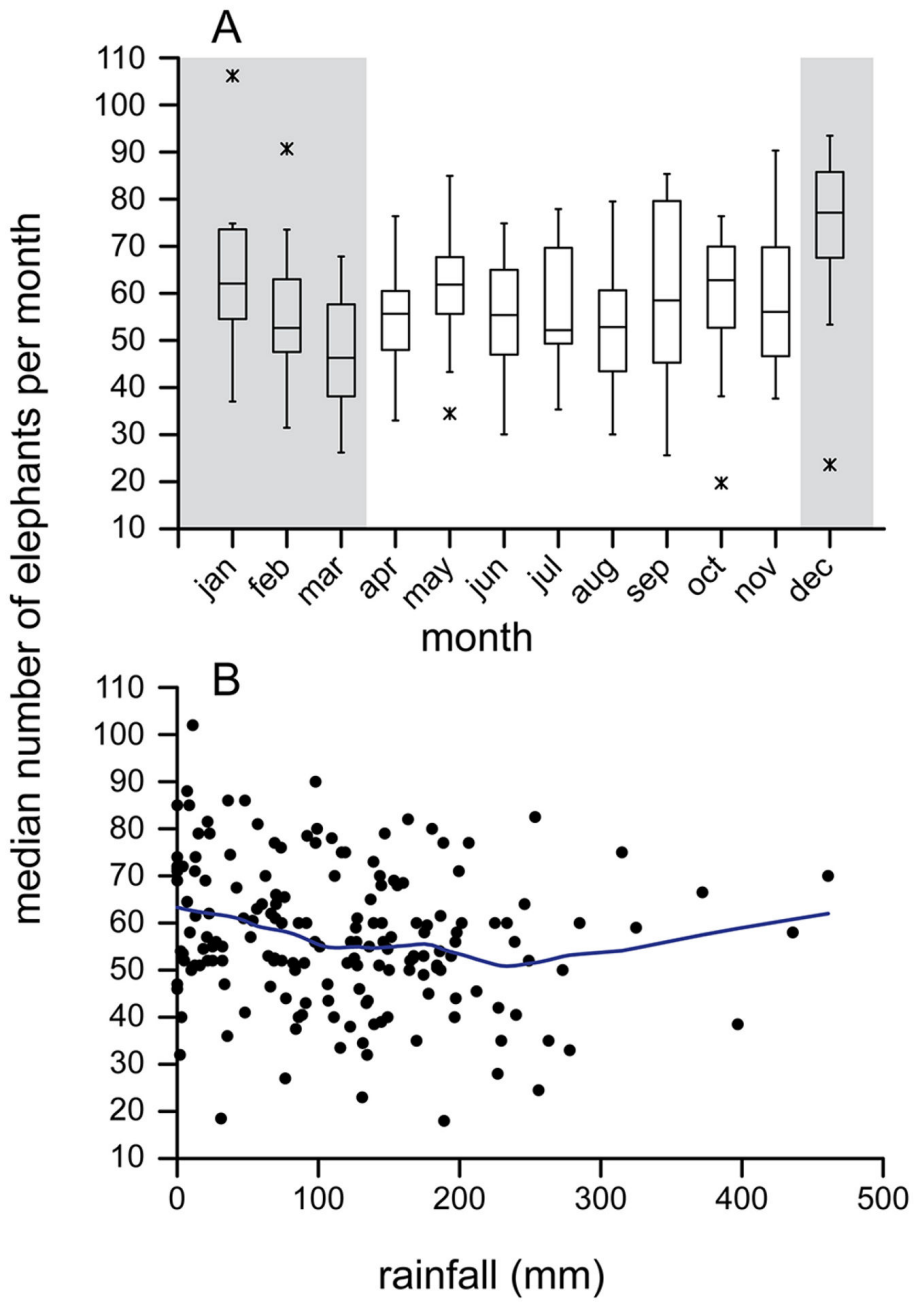
Seasonality and elephant visitation at Dzanga Bai. (A) Wet and dry season patterns of elephant visitation (dry periods indicated by a shaded background; standard boxplot symbology). (B) The correlation between monthly rainfall and visitation by elephants. Solid curve is a LOESS function.

The monthly mean and range of daily maximum scan counts of elephants remained relatively consistent through the study (Figure 7), averaging 58 elephants observed per day (range 0-173). Elephant activity was poorly correlated with rainfall (Figure 8a). We found no significant effect of ‘wet’ vs. ‘dry’ seasons (December-March = ‘dry’) on the average monthly numbers of elephants (GLM model F_s_ = 2.1 for season, p=0.15, n=174 months). A simple correlation between monthly rainfall and elephant numbers was borderline significant (Pearson r = -0.18, p=0.051), indicating fewer elephant with more rain, but explained less than 3% of the variation in elephant numbers (Figure 8b).

## Discussion

### Cumulative Identification and Population Trends

During 20 years of observation at Dzanga, ATK identified nearly 5000 individual elephants, 3138 of which were considered reliably identifiable over any period of absence (observed across > 9 month intervals). The number of elephants visiting Dzanga Bai remained remarkably stable over the 20-year study, at about 1700 individuals each year (averaging roughly 1000 females and 700 males). The sex ratio of adults visiting the Dzanga clearing was skewed significantly toward females. Early in the study females accounted for 56% of identified elephants, however, by the end of the 20 year analysis period, females accounted for 70% of the sampled population. Based on a large sample of the best-known groups, this increased skew was entirely due to a drop in the proportion of adult males by 2010. Although male forest elephants may have slightly higher natural rates of mortality than females, as has been found for *L. africana* [29,30], elsewhere in Africa highly skewed sex ratios are associated with high levels of poaching targeting males [29,30]. The increase in skew over time at Dzanga is similar to that occurring in the Samburu population, which demonstrated remarkably similar changes in skew over a 15 year period after experiencing high poaching pressure [26,27]. This assessment of change in sex ratios over time demonstrates underlying pressures impacting the population that would not necessarily be inferred from assessment of population trends given the relative stability in population size over the 20-year study.

New adults (and families) continuously joined the Dzanga Bai population over the 20-yr study with particularly noticeable upticks in 2002 and 2006-2007 (Figure 2). While some of these new adults were transients (i.e. identified but seen for only a few days) about 40% became regular visitors and represent animals apparently shifting their home ranges to include the Dzanga clearing. Such consistent immigration over many years has not been recorded in long-term studies of elephants in savannah systems [31,32]. We suspect that this immigration is the result of compression, driven by human population growth in areas adjacent to the Dzanga-Ndoki National Park [33] and by commercial logging in neighboring forest tracts, both of which are correlated with increased forest use/access road networks related to expanding infrastructure and result in elephant avoidance [1,9]. It is important to note that the stability in population size may be partially contributed to by these new elephants entering the bai, though focus on core elephants (typically excluding new immigrants) demonstrates similar stability.

There is a long history of elephant hunting in the Dzanga and the greater Congo Basin area as a function of the historically high elephant densities, resulting in the exportation of hundreds of tons of ivory to Europe in the late 19^th^ and early 20^th^ centuries [34,35]. Early in this study hunting was observed to be highly selective: the majority of elephant carcasses encountered were mature males targeted for their tusks (AKT, personal observation). Recently, the hunting of smaller individuals including females and young males has become common, with the focus shifting to include meat as well as ivory (AKT, personal observation). A recent study suggests poaching pressure has caused a dramatic decline in the continental forest elephant population [1]. The relatively low illegal killing rate in Dzanga relative to other forest sites [36] may be a function of the protection afforded the population by the long term monitoring and guard presence at and around the clearing. As such, the relative stability in the population size reported here may be a best-case scenario for the species (relative to results reported for other forest populations [1] and highlights the uniqueness of this site, not only in terms of the long term individual identification study, but due to its relative protection. As such, continued monitoring of the Dzanga population may offer one of our best opportunities to understand forest elephant biology in a setting not severely impacted by humans. 

### Patterns of visitation

The average daily number of elephants seen in the clearing (derived from scan samples) each month remained quite stable through the study (mean=58 elephants/day, SD=16). In addition, we found no biologically relevant correlation between the number of individual elephants visiting the bai and either dry versus wet seasons or actual monthly rainfall. Similar lack of seasonality was reported for M’Beli Bai in northern Republic of Congo [28], which shares the same landscape with Dzanga. A three-year study at Langoué Bai in Gabon reported that more elephants were observed in the short dry season and fewer elephants observed in the long dry season [12]. However, acoustic data from Langoué and three other bais in the same landscape, which assessed elephant numbers over a single annual cycle, showed coincident peaks in elephant activity at the four sites but peaks were not related to rainfall [24]. 

This inter-month and inter-annual consistency in observed elephant numbers is in stark contrast to the pattern observed in savannah elephant populations [26,31]. Within year volatility in savannah systems is related to seasonality while between years variation is driven by cycles of drought or above average rainfall, which strongly influences mortality and reproduction [32,37]. By contrast, the equatorial rainforests of Central Africa offer a much more stable environment, where water is always available (even through short dry seasons). Food resources may be limited (with productivity in the canopy rather than at ground level) with the patchy, dispersed nature of resources being suggested as the major reason that social groupings are considerably smaller in forest elephant societies [38,39]. The observed stability in bai use probably indicates the weak seasonal influences on forest elephant behavior, and it will be important to assess if this is reflected in stability in inter-annual demographic rates. This multi-year study at Dzanga, along with an increasing number of shorter studies at other forest clearings, show that we still have little understanding of the physiological and ecological factors that drive individual elephants to visit forest clearings.

Most individual forest elephants were observed within the Dzanga clearing for only a few days per year and there was little difference between males and females (Table 1). From the diurnal observations compiled here, one would infer that an average female spends 2% of her year in the Dzanga clearing and the average male 1.6%, though variation between individuals and family groups was high with some visiting as often as every few months and others skipping one or more years (Figure 5). Collating these data by month, 70% of 623 males and 59% of 914 females were seen fewer than three months per year and there was no evidence of multi-modality as used to distinguish subpopulations in other systems [26]. The mode of months observed per year was two for both sexes, though females were significantly more likely to be seen in the bai. Because observation periods covered only a small part of the day, and the largest numbers of elephants are present at night [24,40], some visits were undoubtedly missed. Increasing nocturnal activity seems to be characteristic of elephants that have been traumatized by human activity [24,41-43]. Since male forest elephants are primary targets of poaching efforts they may be more likely than females to avoid exposure in open areas during the day, potentially explaining some of the differences in observation patterns. If illegal killing pressure has increased in the system over the course of the study (which is not supported by MIKE data until a killing event that happened in 2013), this pressure differential could contribute to the reported increase in sex ratio skew.

An important unknown question is whether forest elephants spend only very little time in a *specific* clearing but frequent many other sites within their home range. On average, 7-8 months elapsed between successive visits by an individual or family group. This suggests that few elephants remain in close proximity to the Dzanga clearing but rather are ranging long distances in search of food and possibly mating opportunities. In addition, nearly 25% of the population was highly sporadic in their use of Dzanga (intervisit intervals > 1year). It is possible that some forest elephants may be less philopatric than savannah elephants, leading to semi-nomadic space use or continuous range exploration/expansion. Telemetry of four forest elephants over a 7-9 month period in the Dzanga-Ndoki complex [16] showed little distinction between males and females in ranging behavior. These four elephants were tracked visiting five different bais and spent an average of about 130 days/yr within 500m of a bai [16]. Another telemetry study of four elephants collared near a bai in Gabon obtained similar results [12]. Fewer than 2% of the elephants observed at Dzanga averaged more than 30 days per year in the bai, indicating inference on bai use by direct observation during daylight hours may be biased low and highlighting the need for sustained monitoring to collect useful data through individual identification in this system. 

### Social organization in forest elephants

The size and composition of groups entering forest clearings has been used as a proxy for group size in forest elephants [12,19,23,44]. With more than 40,000 groups observed, the most comprehensive summary of social properties of this nature for a single population is provided here. In stark contrast to the all-male groups characteristic of savannah elephants [45], adult male forest elephants overwhelmingly enter bais alone (multi-male groups were observed <0.3% or 105 times). Head et al. [46], using camera traps placed along trails and crossing points in swamps, found that males sometimes associated in groups of two to three, but their occurrence was rare compared to sightings of solitary males. Forest clearings might be the equivalent of male-areas in the savannah [45,47] where males can interact, establish and maintain the dominance hierarchy, and perhaps learn social skills. The size of family groups entering Dzanga clearing averaged 3.0 (SD = 1.2), comparing closely with other studies of forest elephant group size: 3.1 and 3.2 in coastal habitats of Gabon [46,48]; 3.4 bai northern Congo [44]; 3.5 and 3.9 interior forest-savannah mosaic in Gabon, [12,38]; 3.0 bai Gabon [12]. Although group size estimates within the forest are available for only a single study [46], results here support the suggestion that forest elephants are predominantly in nuclear family groups of mothers with dependent offspring. Based on behavioral interactions, forest elephants maintain much larger networks of associations [19,22,49], but how ‘family gatherings’ at forest clearings are orchestrated and whether some sort of extended family cohesion is maintained in the forest at large, remain intriguing but unanswered questions. 

## Conclusions

The reported stability of the Dzanga population over the 20 year study period highlights the uniqueness of this population in respect to current, excessive harvesting pressures on this species. The data reported here represent the most comprehensive information available regarding bai usage by forest elephants, information critical for designing effective monitoring programs based on bai based observations. The initial time investment of approximately four years to characterize the elephant population and the stochastic use patterns of known elephants demonstrates the importance of a long-term commitment to any such study in order to derive useful trend, demographic and behavioral data. The length of the initiation period in this study probably was less a function of the large Dzanga population than due to high variation in visitation intervals and the probability that an individual was actually observed during a visitation bout (e.g. appears in the clearing during an observation period rather than at night, etc.). 

The likelihood that only a fraction of an individual animal’s visits to a clearing can be recorded during daytime-only observation, and the predominance of activity at night even in this very busy and well-protected site, raise substantial challenges for enhancing the value of bai studies. Given the apparent tendency of forest elephants to become more nocturnal when threatened by poaching or other anthropogenic disturbance [24,41-43], consistent monitoring of day versus night activity would be informative [24], particularly if related to independent measures of poaching pressure like MIKE data. Relative day versus night activity can be obtained most efficiently using autonomous acoustic recordings [24], although thermal imaging technology (unlike night-vision equipment) can also provide accurate nocturnal counts (P.H.W. and A.K.T., unpublished data). The high acuity of thermal imaging could potentially allow identification of individuals at night, but would require developing a set of identifying characters completely independent of those used during the day, as many of the latter are either invisible or easily confounded in thermal images (P.H.W. and A.K.T., unpublished data). 

The data collection methods established during the course of this study are best suited to estimate demographic parameters, study fine scale behaviors (and their changes over time), and to monitor the possible impacts of hunting pressure or land use changes on a bai population. Assuming bai use is sporadic across the forest elephant range, short-term studies may be most suited for rapid behavioral assessments (i.e. skittishness of elephants, wounding rates, night versus day use, etc.), with multiple years required to derive population level demographic assessments and metrics sensitive to human induced change.

Contrary to perceptions that high productivity forest systems decrease the importance of movement and migration, the results here indicate that few elephants rely simply on a localized area, supporting results from the limited radio tracking studies of this species. This highlights the importance of conservation efforts focused at the landscape level consisting of numerous bai complexes. 
